# Affordance Realization in Climbing: Learning and Transfer

**DOI:** 10.3389/fpsyg.2018.00820

**Published:** 2018-05-28

**Authors:** Ludovic Seifert, Dominic Orth, Bruno Mantel, Jérémie Boulanger, Romain Hérault, Matt Dicks

**Affiliations:** ^1^CETAPS - EA 3832, Faculty of Sport Sciences, University of Rouen Normandy, Mont-Saint-Aignan, France; ^2^Amsterdam Movement Sciences, Faculty of Behavioural and Movement Sciences, Vrije Universiteit, Amsterdam, Netherlands; ^3^CesamS, UNICAEN, Normandie Université, Caen, France; ^4^Laboratoire CRISTAL, University of Lille 1, Villeneuve-d'Ascq, France; ^5^LITIS, Institut National des Sciences Appliquées de Rouen, Saint-Étienne-du-Rouvray, France; ^6^Department of Sport and Exercise Science, University of Porthsmouth, Portsmouth, United Kingdom

**Keywords:** motor learning, affordance, exploration, perception, climbing

## Abstract

The aim of this study was to investigate how the affordances of an indoor climbing wall changed for intermediate climbers following a period of practice during which hold orientation was manipulated within a learning and transfer protocol. The learning protocol consisted of four sessions, in which eight climbers randomly ascended three different routes of fixed absolute difficulty (5c on the French scale), as fluently as possible. All three routes were 10.3 m in height and composed of 20 hand-holds at the same locations on an artificial climbing wall; only hold orientations were altered: (i) a horizontal-edge route (H) was designed to afford horizontal hold grasping, (ii) a vertical-edge route (V) afforded vertical hold grasping, and (iii), a double-edge route (D) was designed to afford both horizontal and vertical hold grasping. Five inertial measurement units (IMU) (3D accelerometer, 3D gyroscope, 3D magnetometer) were attached to the hip, feet and forearms to analyze the vertical acceleration and direction (3D unitary vector) of each limb and hip in ambient space during the entire ascent. Segmentation and classification processes supported detection of movement and stationary phases for each IMU. Depending on whether limbs and/or hip were moving, a decision tree distinguished four states of behavior: stationary (absence of limb and hip motion), hold exploration (absence of hip motion but at least one limb in motion), hip movement (hip in motion but absence of limb motion) and global motion (hip in motion and at least one limb in motion). Results showed that with practice, the learners decreased the relative duration of hold exploration, suggesting that they improved affordance perception of hold grasp-ability. The number of performatory movements also decreased as performance increased during learning sessions, confirming that participants' climbing efficacy improved as a function of practice. Last, the results were more marked for the H route, while the D route led to longer relative stationary duration and a shorter relative duration of performatory states. Together, these findings emphasized the benefit of manipulating task constraints to promote safe exploration during learning, which is particularly relevant in extreme sports involving climbing tasks.

## Introduction

Research indicates that the realization of a multitude of skillful human behaviors, including throwing (Zhu and Bingham, 2010; Wilson et al., 2016), catching (Peper et al., 1994; Montagne et al., 1999; Zaal and Michaels, 2003), and climbing (Seifert et al., 2014b) are predicated on the accurate perception of affordances (i.e., opportunities for action; Gibson, 1979). The accurate perception of an affordance entails an individual's ability to perceive what the environment offers her or him relative to her or his own abilities (Fajen, 2005; Chemero, 2009; Withagen et al., 2012). For instance, in a seminal study, Warren (1984) reported that despite differences in body size, young adults accurately perceived stairs as no longer climbable in a bipedal fashion, when the step's height exceeded 88% of their lower limb's length (for further considerations, see Konczak et al., 1992; Cesari et al., 2003).

How are affordances perceived? The perception of affordances is believed to rest on the pick-up of information that specify patterns in ambient stimulation, or so-called invariants^1^ (e.g., Gibson, 1979). These patterns are not to be thought of as pre-given structures that would be imposed on passive sensory receptors (as would a picture or movie on a screen), rather they are actively sought after by perceiving individuals (hence the use of *perceptual systems* in place of *senses*; Gibson, 1966; Stoffregen et al., 2017). It follows that, through action, individuals learn to create information to support performatory movements using *exploratory* actions. While the distinction may not be exclusive, it is worth noting that in humans exploring appears to be the nature of actions first used for during the first moments of life (Gibson, 1988).

Exploratory actions can be of two different kinds. First, they may be intrinsic to the act of perceiving. Indeed, since behaviorally relevant characteristics of the human-environment fit are specified in the patterns of stimulation and since these patterns are contingent on the perceiver's motion, then she/he literally (co-)constructs information through her/his actions (Mantel et al., 2015). Second, exploratory actions may be used to seek (new) information (e.g., invariants allowing better performance or efficiency); that is, they may support perceptual learning (i.e., learning to perceive affordances). Such exploratory actions are pervasive in children, playing a core role in development and are no less important in adults for they constitute the basis of their perceptual ability to adapt to new situations and develop new skills (Gibson, 1988). The invariants specifying affordances are contingent on nested information suggesting that postural stability, reaching and grasping a hold are all actions nested within the process of climbing to end of a route—each with potentially specific exploratory actions.

What do information-gathering actions look like? Depriving or restricting participants in their exploration have been showed to hinder affordance perception (e.g., Mark et al., 1990; Mantel et al., 2015) and recalibration (when action capabilities have changed; e.g., Mark et al., 1990; Withagen and Michaels, 2002; Wagman and Van Norman, 2011). For example, not allowing participants to move or requiring the adoption of an awkward stance (with feet together and toes apart) degrades their capacity to judge the maximum height on which they could sit (Mark et al., 1990). Furthermore, when wooden blocks are attached to the foot soles (which changes sitting capability), standing participants leaning against a wall do not exhibit recalibration in their judgments. In contrast, non-leaning participants, and participants allowed to walk between trials, recalibrate judgments to more accurately perceive optimal sitting height. Exploratory actions also seem to be tailored to the affordance to be picked up, and, yet, they may (e.g., Michaels et al., 2007) or may not (e.g., Mark et al., 1990) resemble the action afforded. If perceptual learning is conceived as a process of differentiation (i.e., attending to different, more useful, invariants; e.g., Gibson and Gisbon, 1955) rather than a process of enrichment (i.e., attending to the same cues, but interpreting them in a better way), one may expect a change in exploratory behavior as learning develops. The precision of affordance judgments has been reported to be contingent on the type of exploratory activity used by participants (e.g., Mantel et al., 2015), and participants that improved in their judgments have been observed to exhibit properties in their exploratory activity that non-improving participants do not (e.g., Stoffregen et al., 2005).

Specific to the development of expertise, it has been proposed that skilled performers in domains such as sports, perceive and realize affordances across a larger range and with greater accuracy than novices (Fajen et al., 2009). When considering the skill of climbing, Gibson (1979) suggested that “*slopes between vertical and horizontal afford walking, if easy, but only climbing, if steep and in the latter case the surface cannot be flat; there must be holds for the hands and feet*” (p. 132). Therefore, although a steep 10 m high wall does not afford walking (bipedal locomotion), it may afford climbing (quadrupedal locomotion) for the individual with the required abilities. *Climbability* depends on the relation between the characteristics of an approximately vertical surface and its layout (e.g., holds for the hands and feet) and that of an individual, which constrain her/his reaching, grasping and using holds as the basis for quadrupedal locomotion. Importantly, rock climbing does not only correspond to continuous upward body displacements, but also includes stationary positions dedicated to exploring and grasping surface holds (Pijpers et al., 2006; Sibella et al., 2007), postural regulation (Bourdin et al., 1998, 1999) and route finding (Cordier et al., 1993, 1994). Route finding skill reveals the ability of climbers to adapt to the ever-changing structural and functional features of the climbing wall (Cordier et al., 1993, 1994), in order to explore actions, including opportunities to grasp a hold in a certain way (e.g., crimp, pinch, slope) and to use the hold within a particular coordination mode (e.g., arm crossing or dual grasping on the same hold; Boschker and Bakker, 2002). Therefore, rock climbing is a complex form of locomotion as it involves interspersed periods of perceptual-motor exploration for route finding (Button et al., 2016; Seifert et al., 2017) with combinations of upper and lower limb movements to ascend the surface safely and fluently (Nougier et al., 1993; Boschker et al., 2002; Sibella et al., 2007).

The time spent in exploration, postural regulation and ascent, or more broadly, the time spent stationary or in motion, can be analyzed by quantifying the durations when the hip (as an indicator of the center of mass) is or is not in motion (Billat et al., 1995; Sanchez et al., 2012; Seifert et al., 2013, 2014b). Billat et al. (1995) noted that experienced climbers spent 63% of a route duration stationary and 37% ascending. Pijpers et al. (2006) distinguished between exploratory and performatory movements through the analysis of holds that were and were not used during the ascent. Exploratory movements occurred when grasping actions oriented toward a particular hold did not subsequently lead to the use of that hold during the ascending climb, whereas performatory movements corresponded to hand grasping actions performed with simultaneous ascending hip motion. Using the ratio between touched-grasped holds and used holds, Sibella et al. (2007) reported that skilled climbers tended to touch fewer than three surface holds before *using* the functional hold. Moreover, Nieuwenhuys et al. (2008) showed that exploration for route finding or hold reaching and grasping does not only occur through hand movement, but also through visual exploration when the climber is stationary or regulates her/his posture.

It is plausible that a high number of exploratory movements may reflect low route finding skills in the sense that the climber may not immediately detect hold depths (Nougier et al., 1993) or hold orientations (Seifert et al., 2015) in relation to her/his own characteristics and ability to perform ascending actions. Such suggestion was recently examined through the manipulation of the hold orientations on a climbing route, which invited different grasping actions; namely, horizontal, vertical, and double-edge (i.e., both horizontal and vertical orientation were available) holds (Seifert et al., 2015). The route designed with double-edged holds led the climbers to exploit both a pre-existing behavioral repertoire consisting of a horizontal hold grasping pattern and trunk facing the wall and to explore new behaviors, specifically, vertical hold grasping and trunk facing side-on to the wall (Seifert et al., 2015). These findings indicated that the climbers functionally explored so that they became attuned to the information that specified the different ascending behaviors. In the study of Seifert et al. (2015), functional exploration entailed two aspects: (i) the climber did not use only one part of the body such as the right hand to reach the hold, but she/he used the whole body by adapting the rolling motion of the body to provide support for the pattern necessary to grasp the hold (horizontally or vertically); and (ii) while using the whole body coordination just described, the climber was able to achieve the same performance outcome, for example in terms of route completion and climbing fluency.

Although exploration is proposed to play an important role in practice and development, spending an excessive time stationary for route finding, hold exploration or postural regulation may clearly compromise climbing fluency (Cordier et al., 1993, 1994; Seifert et al., 2014a). Recently, Orth et al. (2017b) suggested the idea that individuals shift toward variables for the perception of affordances that support more fluent climbing, but this hypothesis remains not tested experimentally. Thus, the role that exploration plays in task achievement is not yet fully understood (Seifert et al., 2015). At present, exploration is thought to play a role in climbing but is then believed to decrease once a route is learnt (e.g., climbing fluency improved after six ascents of the same route in Cordier et al., 1993, 1994). However, it is currently unknown how the design of a route (e.g., hold size, distance between holds, hold shape, hold orientation) stimulates exploration during learning and task achievement as previous research (Cordier et al., 1993, 1994, 1996) has not reported the design of the climbing wall (route).

One way to better understand the relation between exploration during learning and task achievement is to vary the route so that learners search for different performatory solutions. Therefore, in the present study, we investigate what affordances an indoor climbing wall offered inexperienced climbers through a learning and transfer protocol with manipulation of the ascending route. The aim of this study was to understand how exploration during learning could help inexperienced climbers to perceive opportunities for action in climbing (i.e., climbing affordances). We hypothesize that, through exploration, climbers learn to pick up informational variables that support behavioral adaptions relative to variations in the design of the climbing route. Specifically, we hypothesize that (i) exploratory behavior should decrease as learning occurs and that (ii) exploratory behavior should be less present in situations affording a single mode of hold grasping rather than multiple modes of hold grasping.

## Methods

### Participants

Eight climbers (five males and three females) with a mean age of 21.0 ± 2.4 years; mean height: 167.0 ± 10.8 cm; mean arm span: 168.4 ± 10.6 cm; mean weight: 57.6 ± 8.9 kg participated in the study. Participants had practiced indoor climbing for 2.2 ± 1.1 h per week for 1.1 ± 0.8 years and had a 5c climbing ability on the French Rating Scale of Difficulty (F-RSD) (Delignières et al., 1993), which represents an intermediate level of performance (Draper et al., 2011) and corresponds to the control stage of motor learning (Newell, 1991). The study was carried out in accordance with the recommendations of the guidelines of the International Committee of Medical Journal Editors and written informed consent from all participants was obtained, in accordance with the Declaration of Helsinki. The protocol was approved by a University local ethics committee. Vulnerable populations (e.g., minors, persons with disabilities) were not involved in the study.

### Protocol

The learning protocol comprised four sessions, each lasting 1 h and separated by 2 days of rest, that required participants to ascend three different grade 5b routes. All participants had 3 min to preview each route prior to climbing and there was a 4-min rest interval between each climb. The order of routes was randomized. Immediately the fourth session, the participants also completed a transfer test, in which a new (fourth) route was climbed.

**Figure 1:**
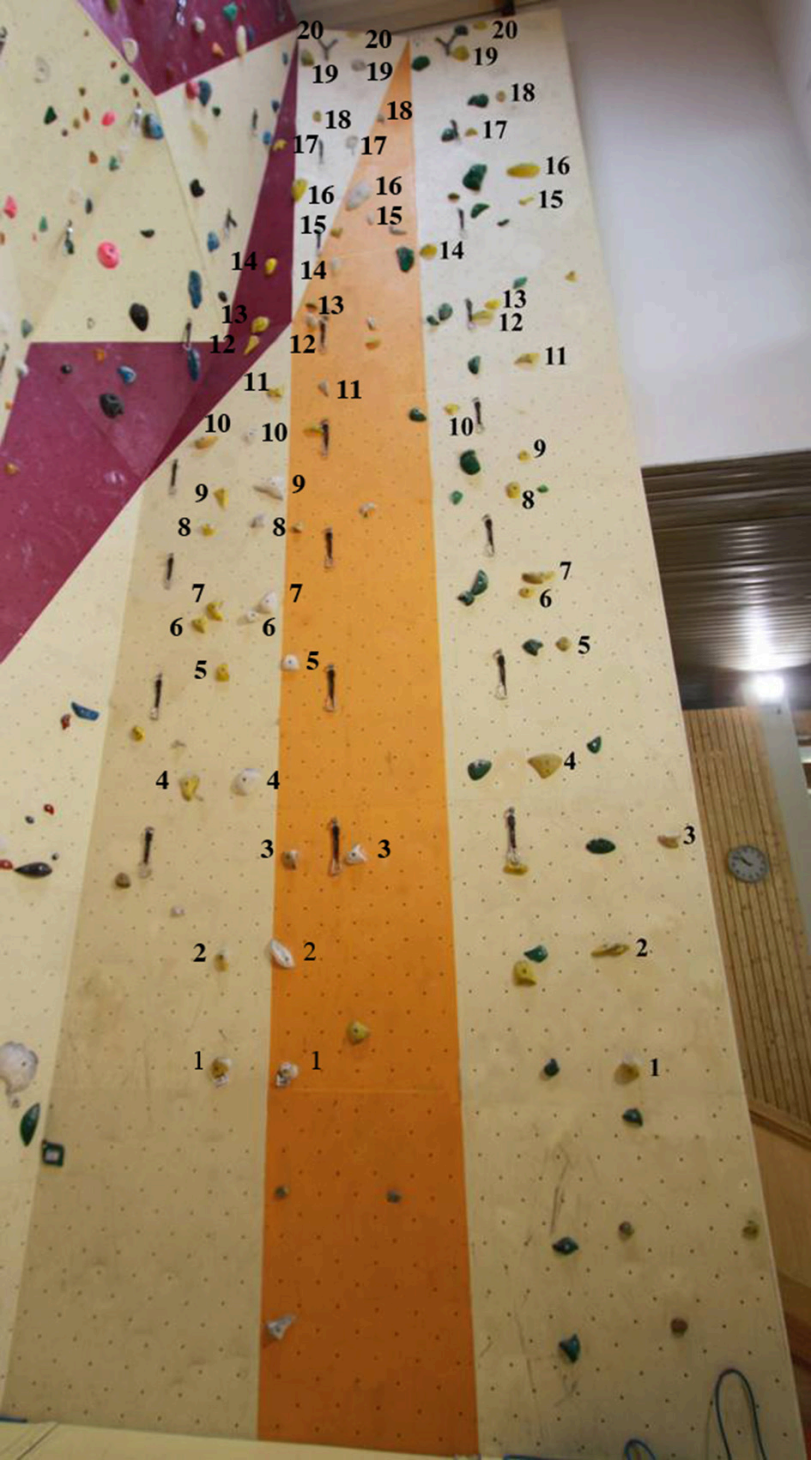
Location of the 20 hand-holds for the three routes. Adapted from Seifert et al. (2015).

Each route was identifiable by color and was set on an artificial indoor climbing wall by three professional certified route setters who ensured that the routes matched an intermediate climbing ability (i.e., physical–technical difficulty grades of 5b on the F-RSD). The three routes had the same height (10.3 m) and they were composed of the same number of hand-holds (20), which were bolted to a flat vertical surface (Figure 1).

**Figure 2:**
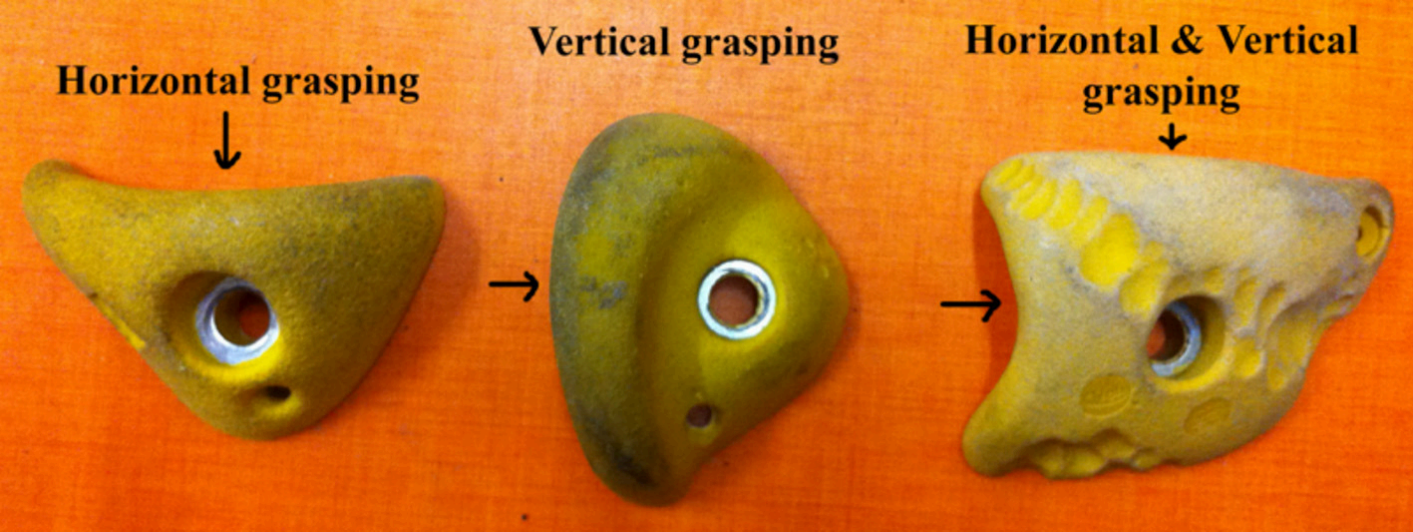
Orientation and shape of the holds for the three routes. The arrow indicates the preferential grasping allowed by the hold. Adapted from Seifert et al. (2015).

The holds were located at the same place on the artificial wall for all routes; only the orientation of the hold was changed: (i) the first route was designed to allow horizontal hold grasping (H), (ii) the second route was designed to allow vertical hold grasping (V), and (iii) the third route was designed to allow dual grasping (D), (i.e., both horizontal and vertical hold grasping) (Figure 2). Furthermore, the route was set to ensure that the footholds invited a vertical grasping pattern, without preventing a horizontal grasping pattern. The difficulty of the route therefore remained the same as the other conditions (i.e., 5b on the F-RSD), but the complexity of the route path and associated holds was higher. Three professional certified route setters confirmed that the routes were of similar difficulty but varied in complexity of route design.

Each route was top-roped, which meant that the route was climbed with the rope anchored above the climber at all times. Each ascent was preceded by 3 min of route preview, which is assumed to be a key climbing performance parameter (Sanchez et al., 2012; Seifert et al., 2017). No instructions were given prior to the route preview to ensure that the opportunity for pre-ascent visual exploration of the climbing route was the same for all participants.

Participants were instructed to self-pace their ascent, with the following task-goal: find a way to climb the wall as *fluently* as possible, without falling down and by minimizing pauses and changes in body direction (Cordier et al., 1993, 1994, 1996; Seifert et al., 2014a). The instructions were not too specific to ensure that climbing actions—and subsequently any exploratory or performatory behaviors—emerged relative to the task constraints of each condition.

Last, a transfer test was designed as a mix of the three previous routes: the first six holds only invited horizontal grasping, the seven next holds only invited vertical grasping, while the last seven holds invited both horizontal and vertical grasping. This route was designed to assess the capability of the climbers to utilize the grasping patterns that they may have developed during the completion of the three practice routes. Thus, the transfer test should be considered as a whole as a new route where an analysis per section is not meaningful (i.e., each section cannot be analyzed separately). Indeed, considering the concept of “nested affordances,” we hypothesized that the holds are not perceived separately (i.e., step by step) but could be perceived as a sequence of possibilities of action (Seifert et al., 2017). It would mean that the current behavior of the climber is linked to where he comes from and where he goes next. Therefore, a fluent climbing would be obtained by fluent transition between holds and not by saccadic displacement resulting from step by step problem solving.

### Data collection

#### Route difficulty and design

The difficulty grade of the route was not given to the participants. After the 3 min of route preview, participants were required to estimate the difficulty grade of each route before the ascent. Moreover, how climbers approached the route, in particular how they perceived hold *grasp-ability* and *use-ability* was determined via a modified version of the *presentation, approach, evaluation* questionnaire (PAE; Sanchez and Dauby, 2009), which focused on the climber's “approach.” During the first and fourth practice sessions, perceived hold *grasp-ability* was assessed with the following questions: (a) I easily perceive the holds dedicated for the feet and for hands, (b) I perceive the best manner to grasp the holds; then, perceived hold *use-ability* was assessed as follows: (a) I easily perceive the movements to do, (b) I easily perceive how to pass from one movement to the next. All answers were given using a 7-point Likert scale (1 = disagree; 7 = fully agree). The estimations of hold grasp-ability and use-ability were then related to behavioral data during the climb.

#### Behavioral data

**Figure 3:**
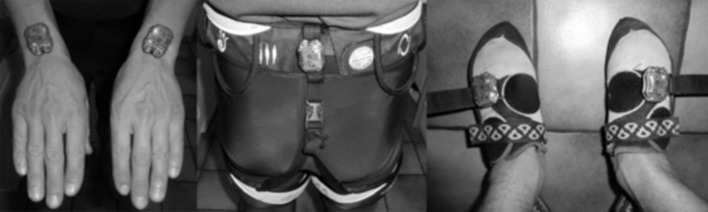
Location of the five IMUs on the body.

Using a novel method developed by Boulanger et al. (2016), we collected data from the four limbs and hip direction (3D vector in Earth reference frame) using inertial measurement units (IMU) located on the wrists, feet, and the hip (Figure 3).

The IMUs combine a tri-axial accelerometer (±8G), a tri-axial gyroscope (1,600°s^−1^) and a tri-axial magnetometer (*MotionPod*, Movea©, Grenoble, France) and have been used in previous research to assess jerk of the hip trajectory during a climbing task (Seifert et al., 2014a). The outlined configuration of wearable IMUs, with North magnetic reference, was utilized in the current study to record movement data, which was sampled at a frequency of 100 Hz. Wireless transmissions from the IMUs to a controller enabled recording of the movement data through *MotionDevTool* software (Movea©, Grenoble, France).

### Data analysis

#### Performance outcome and climbing fluency

The performance outcome of each climb was assessed by the number of falls and the ascent duration. Climbing fluency was assessed through the jerk coefficient, which is an indication of the smoothness of hip trajectory (Seifert et al., 2014a). To determine the jerk of the trajectory, the orientation of the sensor is required, first by removing the component due to gravity, since acceleration is measured in the sensor referential, and second, by determining the angular acceleration. By combining the raw data from the accelerometer, gyroscope and magnetometer, it was possible to compute the orientation of the IMU with respect to the fixed frame of reference of the Earth (magnetic North, East and gravity directions; Madgwick et al., 2011). From this point, the acceleration of the hips was computed in the fixed Earth reference frame, and then used to determine the jerk coefficient (for more details about the method and equations, see Seifert et al., 2014a).

For a trajectory *x*^*GF*^ ∈ *C*^3^([*O, T*]) representing the 3D path of the climber's hips in the time interval [*O, T*] with respect to the ground frame (GF), the jerk $J_{x^{GF}}$ was defined as:

$$\begin{array}{r} J_{x^{GF}}\left(T\right)=C\overset{T}{\underset{0}{\int }}||{\overset{\&}{x}}_{s}^{GF}|{|}^{2}ds \end{array}$$

where *C* is a normalization constant used to make the quantity dimensionless (Hogan and Sternad, 2009), depending on the height and the total climbing time T. In practice, instead of computing $x_{t}^{GF}$ (position on the wall) from $a_{t}^{GF}$ with successive integrations, the term ${\overset{\ldots }{x}}_{s}^{GF}$ was replaced by ${\overset{}{a}}_{t}^{GF}$. By derivation of $a_{t}^{GF}$, the constant gravity acceleration was removed, leaving only the hip acceleration component.

#### Climbing affordances

A multiple sensors-based analysis was conducted to detect the different actions of the climber (Boulanger et al., 2016). Using the data derived from the multiple sensors, five climbing states were identified depending on whether the limbs and/or hip were moving or stationary (Boulanger et al., 2016): stationary (i.e., all limbs were immobile and the hip was immobile), postural regulation (i.e., all limbs were immobile and the hip was moving), hold exploration (i.e., at least one limb was moving and the hip was immobile), hold change (i.e., last hold grasping before body motion), performatory movements (i.e., at least one limb was moving and the hip was moving). The climbing states identified by the method of Boulanger and colleagues were then amended for the purpose of the current study as follows: (i) The *stationary* state represented the state when the climber was not moving at all. She or he might have been resting due to fatigue or might have been looking at the route to determine a subsequent climbing path; (ii) The *postural regulation* state represented an adjustment of the center of mass of the climber while the limbs remained on the same holds. This comprised a body rotation to be able to catch a hold that would not have been reachable from a previous body configuration; (iii) The *hold exploration* state corresponded to the hip remaining stationary while the climber modified the position and orientation of the hands/feet on a hold or performed repetitive movements with one limb to determine which hold to use for the next body motion. These two ways of exploring the holds, which have been characterized as *hold exploratory movements* by Pijpers et al. (2006), were assumed to correspond to a climber's evaluation of the *grasp-ability* of a hold; (iv) The *hold change* state corresponded to the hip remaining stationary while the climber changed the grasped hold before body motion (i.e., *hold transitional* movement); and (v) The *performatory* state represented the state when the climber's hip was moved with concurrent motion of at least one limb, which has been characterized as a hold *performatory movement* by Pijpers et al. (2006), and were assumed to provide an indication of a hold's *use-ability* for the climber.

The relationship between exploratory and performatory movements considered above was used to study how climbers attuned to affordances; that is, how they perceived the climbability of the environment (Boschker and Bakker, 2002; Boschker et al., 2002; Pijpers et al., 2006).

Collectively, the time spent *stationary* and performing *postural regulation* capture a part of exploratory activity that could characterize “route finding” skill, defined as the path followed to perform an ascending climb (Cordier et al., 1993, 1994). In the current study, the “hold change” state was distinguished from the “hold exploration” state because although those limb movements could be exploratory, their results were directly followed by hold utilization. In that case they would be exploratory movements but more skilled because more targeted than others. Interestingly, one could imagine that perceptual learning should not only lead to a decrease of the amount of limb movements performed to explore (i.e., “hold exploration”), but should in fact also consist of a transition from general exploratory behavior (“hold exploration”) to more targeted exploratory behavior (thus more often successful, hence often categorized as “hold change”). Thus, “hold change” state could be observed not to decrease with practice. But, at the same time, climbers might also move from a sequential patterning of their movement (e.g., limbs then trunk) to more coordinated movements (yielding a greater fluency), leading to targeted exploratory behavior being categorized as perfomatory.

We hypothesized that the changes in the relative duration (expressed as a percentage relative to the whole climb duration), the number of occurrence of each state (stationary, postural regulation, hold exploration, hold change and performatory movements) were measured during the learning sessions and transfer test. Given our hypothesis that exploratory behavior should decrease as learning occurs, we expected to observe a decrease in the relative duration and the number of occurrences in the stationary and hold exploration states across the practice sessions. If the amount of practice was sufficient, positive transfer can be expected, which means that the relative duration and the number of occurrences in these exploratory states in the transfer test would be similar or lower than in the last training session. Conversely, if the amount of practice was not enough, negative transfer can be expected, which means that the relative duration and the number of occurrences in these exploratory states in the transfer test would be higher than in the last training session. As regards our hypothesis about route design, we expected to observe a lower relative duration and number of occurrences in the stationary and hold exploration states in the H and V routes as compared to the D route.

### Statistical analysis

According to the previous hypotheses, the *effects of practice* and *route design* were analyzed by separate two-way repeated measures ANOVAs (practice across four sessions and climbing wall design across three different routes) for the five states of activity (i.e., number of events and relative duration of stationary, postural regulation, hold exploration, foot or hand changes between two holds, body motion), the number of falls, the ascent duration and the jerk coefficient. Effects of practice and route design were also analyzed by separate two-way repeated measures ANOVAs (practice across first and fourth sessions; and climbing wall design across three different routes) for the perception of the route difficulty (via the four questions of the PAE questionnaire). Sphericity in the repeated measures design was verified with the Mauchly test (Winter et al., 2001). When the assumption of sphericity was not met, the significance levels of *F*-ratios were adjusted according to the Greenhouse-Geisser procedure. Then, False Discovery Rate (FDR) correction across all the ANOVA condition main effects was done according to Benjamini and Hochberg (1995). Last, *post-hoc* pairwise conditions comparison tests were applied and family-wise error rate was controlled by applying a Bonferroni correction of the *p*-value (Howell, 2002).

*Skill transfer* was analyzed for the same dependent variables using one-way repeated measures ANOVA and simple contrast tests. Planned contrast tests were used to examine how practice on *known routes* affected performance on *new routes*, by comparing the fourth session of each route with the transfer test. This contrast test is interesting because as suggested in the previous section, the amount of practice can have an effect on exploration both for route finding and hold grasping. Therefore, positive effects of exploration would mean that no significant differences in climbing fluency and exploratory behavior would emerge between the fourth session and the transfer test. Partial eta squared ($\eta_{\text{P}}{}^{2}$) statistics were calculated as an indicator of effect size, considering that $\eta_{\text{P}}{}^{2}$ = 0.01 represents a small effect, $\eta_{\text{P}}{}^{2}$ = 0.06 represents a medium effect and $\eta_{\text{P}}{}^{2}$ = 0.15 represents a large effect (Cohen, 1988). All tests were performed using IBM SPSS Statistics 20.0 (1989–2011), with a level of statistical significance fixed at *p* < 0.05. Except where otherwise indicated, all numerical values in parentheses correspond to the mean and standard deviation.

## Results

### Effect of route design

#### Performance outcome and climbing fluency

Significant effect of route design occurred for the number of falls [*F*_(2, 6)_ = 5.79, *p* = 0.045, $\eta_{\text{P}}{}^{2}$ = 0.79], ascent duration [*F*_(1.39, 6)_ = 5.47, *p* = 0.048, $\eta_{\text{P}}{}^{2}$ = 0.77] and jerk coefficient [*F*_(1.44, 10.13)_ = 5.84, *p* = 0.027, $\eta_{\text{P}}{}^{2}$ = 0.45]. *Post-hoc* tests revealed that a significantly higher number of falls occurred for the D route (4.4 ± 0.7 falls) than the H route (1.1 ± 0.7 falls, *p* = 0.043) and the V route (0.6 ± 0.5 falls, *p* = 0.039). *Post-hoc* tests emphasized significantly longer ascent duration for the D route (114.5 ± 11.3 s) than for the H route (83.5 ± 8.0 s, *p* = 0.003) but no difference occurred with the V route (96.6 ± 9.4 s). Finally, *post-hoc* tests showed significantly higher jerk coefficient values (i.e., a lower fluency) for the V route (1.65 × 10^13^ ± 5.81 × 10^11^, *p* = 0.032) and for the D route (2.68 × 10^13^ ± 9.74 × 10^11^, *p* = 0.028) compared to the H route (3.29 × 10^12^ ± 1.21 × 10^11^).

#### Climbing affordances

Globally, the D route comprised significantly more occurrences of each climbing state (i.e., higher number of periods spent stationary, performing postural regulation, hold exploration, hold change and performatory movements) than the H route (Table 1). Moreover, the relative time spent in a stationary state was significantly longer and the relative time spent in the performatory state was significantly shorter in the D route than in the H route (Table 1).

**Table 1:** Effect of route design on the climbing states (i.e., number of events and total relative duration of the state expressed in % of the whole ascent duration).

**Route**	**Number**	**Relative duration (%)**
**IMMOBILITY**		
Horizontal	19.7 ± 5.0	26.1 ± 5.7
Vertical	27.8 ± 6.2***p = 0.012	31.3 ± 5.1
Dual	30.3 ± 4.3*p = 0.001	34.2 ± 4.0*p = 0.026
ANOVA *F*-value	*F*_(1.18, 8.29)_ = 9.76	*F*_(2, 6)_ = 6.84
*p*-value	0.001	0.027
$\eta_{\text{P}}{}^{2}$	0.92	0.66
**POSTURAL REGULATION**		
Horizontal	11.8 ± 1.4	5.4 ± 1.0
Vertical	16.9 ± 2.3***p = 0.047	5.8 ± 1.0
Dual	19.4 ± 2.3*p = 0.007	6.9 ± 1.2
ANOVA *F*-value	*F*_(2, 6)_ = 10.77	
*p*-value	0.01	
$\eta_{\text{P}}{}^{2}$	0.78	
**HOLD EXPLORATORY MOVEMENTS**		
Horizontal	7.8 ± 2.5	10.6 ± 1.6
Vertical	10.9 ± 3.3	12.0 ± 1.8
Dual	11.3 ± 2.6*p = 0.016	11.2 ± 1.4
ANOVA *F*-value	*F*_(2, 6)_ = 8.64	
*p*-value	0.017	
$\eta_{\text{P}}{}^{2}$	0.74	
**HOLD CHANGE**		
Horizontal	6.3 ± 1.2	5.7 ± 1.1
Vertical	8.4 ± 1.6	6.3 ± 0.7
Dual	10.0 ± 1.0*p = 0.004	8.3 ± 0.6
ANOVA *F*-value	*F*_(2, 6)_ = 18.52	
*p*-value	0.003	
$\eta_{\text{P}}{}^{2}$	0.86	
**PERFORMATORY MOVEMENTS**		
Horizontal	16.7 ± 1.4	52.2 ± 7.3
Vertical	22.6 ± 2.6	44.4 ± 6.3
Dual	25.3 ± 2.5*p = 0.005	39.4 ± 4.7*p = 0.017
ANOVA *F*-value	*F*_(2, 6)_ = 13.93	*F*_(2, 6)_ = 6.75
*p*-value	0.006	0.029
$\eta_{\text{P}}{}^{2}$	0.82	0.69

* Post-hoc tests showing significant differences between the Dual route and the Horizontal route.

*** *Post-hoc tests showing significant differences between the Vertical route and the Horizontal route*.

#### Perception of route approach

A significant effect of route design occurred on the perception of route approach for question 1 (i.e., perception of the holds dedicated for the feet and for hands) [*F*_(2, 6)_ = 5.65, *p* = 0.047, $\eta_{\text{P}}{}^{2}$ = 0.73], question 2 (i.e., perception of the best manner to grasp the holds) [*F*_(2, 6)_ = 5.72, *p* = 0.044, $\eta_{\text{P}}{}^{2}$ = 0.77], question 3 (i.e., perception of the movements to do) [*F*_(2, 6)_ = 5.77, *p* = 0.043, $\eta_{\text{P}}{}^{2}$ = 0.78] and question 4 (i.e., perception of how to pass from one movement to the next) [*F*_(2, 6)_ = 7.61, *p* = 0.039, $\eta_{\text{P}}{}^{2}$ = 0.81]. In particular, contrast test showed that the D route was perceived significantly harder than the H route concerning question 1 [D route: 3.75 ± 0.45 vs. H route: 4.69 ± 0.48; *F*_(2, 6)_ = 11.66, *p* = 0.011, $\eta_{\text{P}}{}^{2}$ = 0.62], question 2 [D route: 3.25 ± 0.49 vs. H route: 4.69 ± 0.32; *F*_(2, 6)_ = 6.63, *p* = 0.039, $\eta_{\text{P}}{}^{2}$ = 0.55], question 3 [D route: 3.56 ± 0.41 vs. H route: 4.44 ± 0.44; *F*_(2, 6)_ = 7.04, *p* = 0.033, $\eta_{\text{P}}{}^{2}$ = 0.51] and question 4 [D route: 3.37 ± 0.41 vs. H route: 4.12 ± 0.39; *F*_(2, 6)_ = 9.02, *p* = 0.02, $\eta_{\text{P}}{}^{2}$ = 0.56].

#### Effect of practice and transfer of learning

##### Performance outcome and climbing fluency

The two-way repeated measure ANOVA showed that the number of falls, ascent duration and jerk coefficient significantly decreased with practice (Table 2); *post-hoc* tests highlighted that these differences occurred between practice 1 vs. practices 3 and 4 (Table 2). No significant interaction was found between route design and practice effects.

**Table 2:** Effect of practice on number of falls, ascent duration and jerk coefficient.

**Trial**	**Number of falls**	**Ascent duration (s)**	**Jerk coefficient**
1	4.1 ± 1.5	120.8 ± 13.8	3.07 × 10^13^ ± 1.28 × 10^12^
2	2.7 ± 1.1	100.9 ± 10.2	1.73 × 10^13^ ± 9.71 × 10^11^
3	1.4 ± 0.9*p = 0.034	87.4 ± 8.8*p = 0.026	9.94 × 10^12^ ± 4.71 × 10^11^ *p = 0.023
4	0*p = 0.01	84.6 ± 6.3*p = 0.021	4.21 × 10^12^ ± 2.78 × 10^11^ *p = 0.02
ANOVA *F*-value	*F*_(3, 6)_ = 5.66	*F*_(3, 6)_ = 16.65	*F*_(1.39, 10.10)_ = 5.76
*p*-value	0.047	0.04	0.031
*$\eta_{\text{P}}{}^{2}$*	0.77	0.85	0.52

* *Post-hoc tests showing significant differences with the first trial*.

No falls were observed during the fourth climb on each route, although 5.5 ± 2.7 falls did occur during the transfer test [*F*_(1, 7)_ = 6.95, *p* = 0.024, $\eta_{\text{P}}{}^{2}$ = 0.57]. Moreover, the one-way repeated measure ANOVA showed a significant effect of transfer on ascent duration [*F*_(1.25, 8.75)_ = 5.76, *p* = 0.035, $\eta_{\text{P}}{}^{2}$ = 0.46]; the simple contrast tests highlighted longer ascent duration on the transfer test (132.7 ± 37.1 s) than on the fourth climb of the H route (75.5 ± 19.4 s) [*F*_(1, 7)_ = 9.88, *p* = 0.016, $\eta_{\text{P}}{}^{2}$ = 0.59], while no significant difference was found between the transfer test and the fourth climb of the V route (92.4 ± 33.9 s) and D route (94.3 ± 25.1 s). Finally, the one-way repeated measure ANOVA showed a significant effect of transfer on jerk coefficient [*F*_(1.05, 7.35)_ = 5.88, *p* = 0.026, $\eta_{\text{P}}{}^{2}$ = 0.48]; the simple contrast tests highlighted significantly greater jerk coefficient during the transfer test (5.19 × 10^13^ ± 2.32 × 10^12^) in comparison with the fourth climb of the H route (7.05 × 10^11^ ± 2.17 × 10^10^), [*F*_(1, 7)_ = 5.91, *p* = 0.042, $\eta_{\text{P}}{}^{2}$ = 0.51], while no significant difference occurred between the transfer test and the fourth climb of the V route (6.91 × 10^12^ ± 4.01 × 10^11^) and D route (5.03 × 10^12^ ± 3.29 × 10^11^).

##### Climbing affordances

With practice, the climbers significantly decreased the number of occurrences and the relative duration of hold exploration across all routes (Table 3).

**Table 3:** Effect of practice on the climbing states (i.e., number of events and total relative duration of the state expressed in % of the whole ascent duration).

**Trial**	**Number**	**Relative duration (%)**
**IMMOBILITY**		
1	27.9 ± 5.7	31.6 ± 4.5
2	28.4 ± 5.6	28.2 ± 4.8
3	22.5 ± 4.6	32.0 ± 5.0
4	21.3 ± 3.7*p = 0.028	30.3 ± 5.9
ANOVA *F*-value	*F*_(3, 6)_ = 7.94	
*p*-value	0.022	
$\eta_{\text{P}}{}^{2}$	0.62	
**POSTURAL REGULATION**		
1	18.0 ± 3.2	6.2 ± 1.6
2	17.3 ± 3.3	7.4 ± 2.1
3	15.3 ± 1.6	5.0 ± 0.9
4	13.6 ± 1.4	5.4 ± 1.1
**HOLD EXPLORATORY MOVEMENTS**		
1	11.0 ± 2.4	13.3 ± 1.8
2	10.6 ± 3.2	11.0 ± 1.9
3	8.9 ± 2.7	9.3 ± 2.1
4	9.6 ± 2.7	8.1 ± 2.2*p = 0.037
ANOVA *F*-value		*F*_(3, 6)_ = 7.17
*p*-value		0.027
$\eta_{\text{P}}{}^{2}$		0.66
**HOLD CHANGE**		
1	8.9 ± 1.4	6.5 ± 0.8
2	7.1 ± 1.1	5.6 ± 0.7
3	9.2 ± 1.4	7.8 ± 1.1
4	8.7 ± 1.3	7.2 ± 0.9
**PERFORMATORY MOVEMENTS**		
1	23.2 ± 3.5	42.4 ± 5.5
2	22.6 ± 3.2	47.6 ± 5.9
3	21.4 ± 1.2	45.9 ± 4.9
4	19.0 ± 1.3*p = 0.025	45.5 ± 6.1
ANOVA *F*-value	*F*_(3, 6)_ = 7.08	
*p*-value	0.029	
$\eta_{\text{P}}{}^{2}$	0.65	

* *Post-hoc tests showing significant differences with the first trial*.

**Figure 4:**
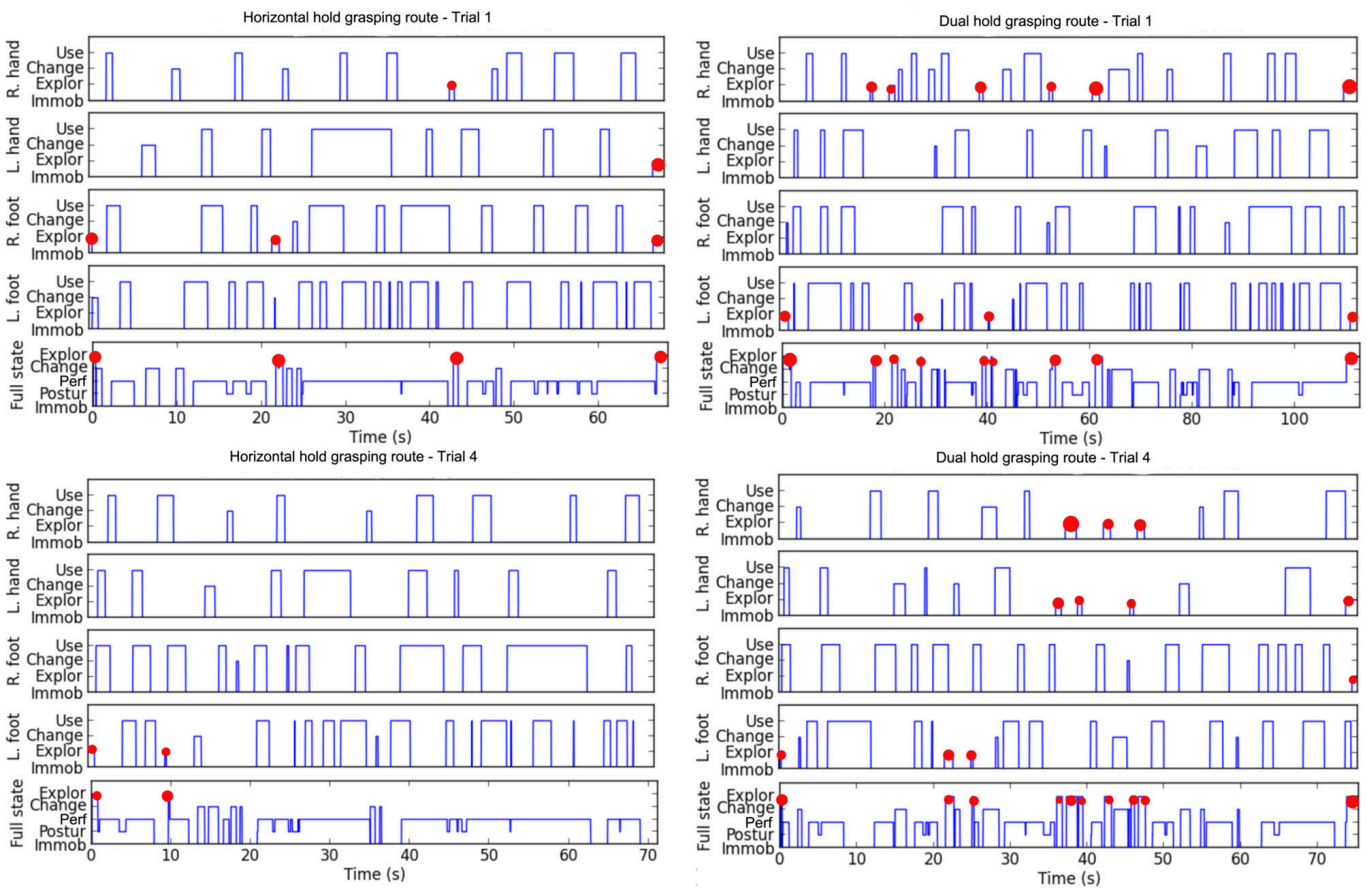
Example for one participant of the climbing states time series for the right hand (R. hand), the left hand (L. hand), the right foot (R. foot), the left foot (L. foot) and the full body state based on the decision tree designed by Boulanger et al. (2016). The four panels exemplified (by red dots) the lower hold exploratory movements on the H route **(Left)** than on the Dual route **(Right)**, and the decrease of hold exploratory movements from trial 1 **(Top)** to trial 4 **(Down)**.

Figure 4 exemplified for one participant the lower hold exploratory movements for H route than for D route, and the decrease of hold exploratory movements from trial 1 to trial 4.

**Figure 5:**
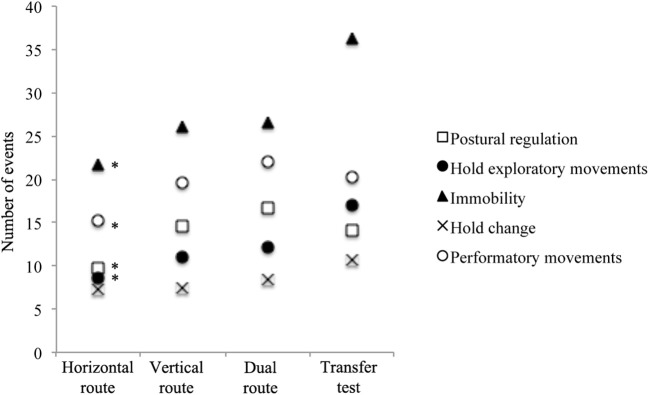
Differences between the transfer test and the three routes performed during the learning protocol concerning the number of events of each of the five climbing states. *Simple contrast tests showing significant differences between the transfer test and the fourth session of each route.

**Figure 6:**
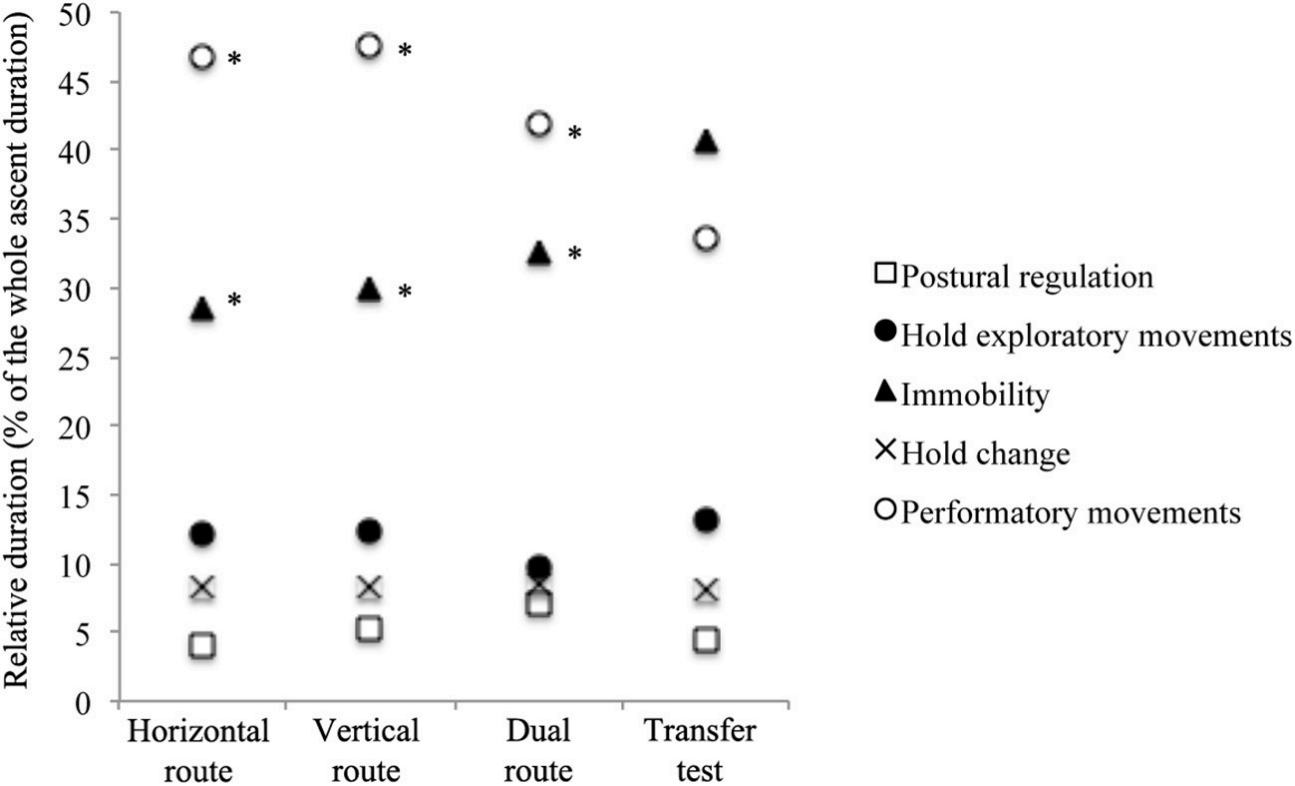
Differences between the transfer test and the three routes performed during the learning protocol concerning the relative duration of each of the five climbing states. *Simple contrast tests showing significant differences between the transfer test and the fourth session of each route.

The one-way repeated measure ANOVA showed a significant effect of transfer on the number of occurrences of exploration [*F*_(1.34, 9.43)_ = 6.21, *p* = 0.028, $\eta_{\text{P}}{}^{2}$ = 0.64], postural regulation [*F*_(3, 5)_ = 30.98, *p* = 0.001, $\eta_{\text{P}}{}^{2}$ = 0.95], stationary position [*F*_(3, 5)_ = 6.63, *p* = 0.027, $\eta_{\text{P}}{}^{2}$ = 0.70] and performatory movements [*F*_(3, 5)_ = 6.09, *p* = 0.031, $\eta_{\text{P}}{}^{2}$ = 0.78]. The simple contrast tests highlighted that the transfer test exhibited a significantly higher number of occurrences of exploration [*F*_(1, 7)_ = 7.47, *p* = 0.026, $\eta_{\text{P}}{}^{2}$ = 0.62], postural regulation [*F*_(1, 7)_ = 21.49, *p* = 0.002, $\eta_{\text{P}}{}^{2}$ = 0.75], stationary [*F*_(1, 7)_ = 7.27, *p* = 0.030, $\eta_{\text{P}}{}^{2}$ = 0.61] and performatory movements [*F*_(1, 7)_ = 6.49, *p* = 0.038, $\eta_{\text{P}}{}^{2}$ = 0.48] than the H route's fourth training session (Figure 5). The simple contrast tests yielded no significant difference for the V and D routes (Figure 5). Finally, as observed for the route design effect, the one-way repeated measure ANOVA showed a significant effect of transfer on the relative duration of performatory movements [*F*_(3, 5)_ = 10.79, *p* = 0.013, $\eta_{\text{P}}{}^{2}$ = 0.87] and stationary position [*F*_(3, 5)_ = 6.85, *p* = 0.036, $\eta_{\text{P}}{}^{2}$ = 0.74]. The simple contrast tests showed that a significantly shorter relative duration was dedicated to performatory movements during the transfer test than during the fourth training session of the H route [*F*_(1, 7)_ = 13.58, *p* = 0.011, $\eta_{\text{P}}{}^{2}$ = 0.66], the V route [*F*_(1, 7)_ = 14.66, *p* = 0.006, $\eta_{\text{P}}{}^{2}$ = 0.68] and the D route [*F*_(1, 7)_ = 12.98, *p* = 0.009, $\eta_{\text{P}}{}^{2}$ = 0.65] (Figure 6). Moreover, the simple contrast tests showed a significantly longer relative duration of the stationary position during the transfer test than during the fourth training session of the H route [*F*_(1, 7)_ = 11.39, *p* = 0.012, $\eta_{\text{P}}{}^{2}$ = 0.62], the V route [*F*_(1, 7)_ = 9.38, *p* = 0.023, $\eta_{\text{P}}{}^{2}$ = 0.52], and the D route [*F*_(1, 7)_ = 7.15, *p* = 0.032, $\eta_{\text{P}}{}^{2}$ = 0.47] (Figure 6).

##### Perception of route approach

With practice, the climbers perceived the route approach to be significantly more difficult at session 1 (S1) than at session 4 (S4), for question 1 [S1 = 3.62 ± 0.48 vs. S4 = 4.67 ± 0.43; *F*_(1, 7)_ = 32.41, *p* = 0.001, $\eta_{\text{P}}{}^{2}$ = 0.82], question 2 [S1 = 3.5 ± 0.32 vs. S4 = 4.58 ± 0.42; *F*_(1, 7)_ = 10.65, *p* = 0.014, $\eta_{\text{P}}{}^{2}$ = 0.61], question 3 [S1 = 3.16 ± 0.28 vs. S4 = 4.71 ± 0.51; *F*_(1, 7)_ = 23.09, *p* = 0.002, $\eta_{\text{P}}{}^{2}$ = 0.77], and question 4 [S1 = 2.83 ± 0.31 vs. S4 = 4.54 ± 0.41; *F*_(1, 7)_ = 70.46, *p* = 0.0001, $\eta_{\text{P}}{}^{2}$ = 0.91]. There was no interaction between the effect of practice and route design, suggesting that the approach of the D route was always perceived harder than the H route. Moreover, as observed for the route design effect, the transfer test was perceived as being significantly more difficult than the three other routes across all four questions (Table 4).

**Table 4:** Differences between the transfer test and the three routes performed during the learning protocol concerning the perception of the route approach.

**Route**	**Question 1**	**Contrast tests**
Horizontal	5.2 ± 1.2*	*F*_(1, 7)_ = 49.0, *p* = 0.0001, η_P_^2^ = 0.87
Vertical	4.5 ± 1.3*	*F*_(1, 7)_ = 28.0, *p* = 0.001, η_P_^2^ = 0.81
Dual	4.2 ± 1.2*	*F*_(1, 7)_ = 5.73, *p* = 0.048, η_P_^2^ = 0.45
Transfer	3.5 ± 1.0	
ANOVA *F*-value	*F_(_*_3, 5)_ = 19.1	
*p*-value	0.004	
$\eta_{\text{P}}{}^{2}$	0.92	
**Route**	**Question 2**	**Contrast tests**
Horizontal	5.3 ± 0.9*	*F*_(1, 7)_ = 37.3, *p* = 0.0001, η_P_^2^ = 0.84
Vertical	4.4 ± 1.3*	*F*_(1, 7)_ = 10.1, *p* = 0.007, η_P_^2^ = 0.68
Dual	4.2 ± 1.3*	*F*_(1, 7)_ = 7.68, *p* = 0.03, η_P_^2^ = 0.52
Transfer	3.2 ± 1.3	
ANOVA *F*-value	*F_(_*_3, 5)_ = 8.96	
*p*-value	0.019	
$\eta_{\text{P}}{}^{2}$	0.84	
**Route**	**Question 3**	**Contrast tests**
Horizontal	5.0 ± 1.6*	*F*_(1, 7)_ = 22.4, *p* = 0.002, η_P_^2^ = 0.76
Vertical	4.8 ± 1.5*	*F*_(1, 7)_ = 22.8, *p* = 0.002, η_P_^2^ = 0.76
Dual	4.4 ± 1.5*	*F*_(1, 7)_ = 10.7, *p* = 0.014, η_P_^2^ = 0.61
Transfer	3.0 ± 1.1	
ANOVA *F*-value	*F_(_*_3, 5)_ = 7.3	
*p*-value	0.028	
$\eta_{\text{P}}{}^{2}$	0.81	
**Route**	**Question 4**	**Contrast tests**
Horizontal	4.8 ± 1.0*	*F*_(1, 7)_ = 63.0, *p* = 0.0001, η_P_^2^ = 0.90
Vertical	4.5 ± 1.2*	*F*_(1, 7)_ = 22.9, *p* = 0.002, η_P_^2^ = 0.77
Dual	4.4 ± 1.5*	*F*_(1, 7)_ = 10.7, *p* = 0.014, η_P_^2^ = 0.61
Transfer	3.0 ± 1.3	
ANOVA *F*-value	*F_(_*_3, 5)_ = 20.6	
*p*-value	0.003	
$\eta_{\text{P}}{}^{2}$	0.93	

* significant different with transfer test.

*Simple contrast tests showing significant differences between the transfer test and the fourth session of each route*.

## Discussion

The aim of the present study was to investigate the role of exploratory behavior in the perceptual attunement of climbing affordances. This was achieved by simultaneously investigating how exploratory behaviors evolved during learning, and how exploratory behaviors differed as a function of the climbers' mastery of the different action modes required across climbing routes with different holds designs. There were therefore two primary perquisites to ascertain for our study: first, whether the participants' performance differed across climbing routes, and second, whether participants' performance improved across learning sessions.

### Effect of route design

The results revealed a significant effect of the route design as (i) the H and V routes resulted in higher performance outcomes (i.e., less falls and shorter ascent duration) than the D route, and (ii) H route yielded a higher climbing fluency (i.e., lower jerk coefficient) than the D and V routes. The absence of an interaction effect between route design and practice indicated that despite initial differences in performance and climbing fluency levels between the route designs, the way that performance improved with practice did not differ for the respective routes.

In order to explain the changes in performance outcomes between the routes, our main hypothesis was that exploratory and stationary behaviors would decrease as a function of one's mastery of the action mode required for climbing each route. In other words, we hypothesized that more complex route designs would afford less to the learners due to their lower abilities to grasp and to use the holds. The results of the PAE questionnaire suggested that when complex action modes were solicited by the route design, the learners less frequently *perceived the hold grasp-ability* (i.e., which and how to grasp the holds). Specifically, in our study the climbers actualized the vertical hold grasping pattern less frequently than the horizontal hold grasping pattern.

Routes V and D also led to longer relative durations of stationary moments than route H. The longer relative duration of the stationary action mode could reflect periods of visual exploration that were utilized to actively search the climbing wall (Button et al., 2016; Orth et al., 2016; Seifert et al., 2017) and thus might reflect “route finding” skill (Cordier et al., 1993, 1994). Route finding requires the determination of a spatial pathway that enables climbers to link movements in time across a landscape of nested affordances in order to reduce stationary periods during the ascent (Cordier et al., 1993, 1994; Orth et al., 2016). The current results are in line with previous research, which has revealed that when climbers of various skill levels ascend a route, the less skilled climbers use a hold-to-hold approach as they exhibit simple grasping patterns (i.e., dual-hand grasping on a hold) whereas skilled climbers exploit more complex grasping patterns (i.e., arm crossing between holds; see Boschker and Bakker, 2002 for more details), which resonates with the proposal that skilled climbers perceive climbing routes as a *landscape of affordances* (Bruineberg and Rietveld, 2014; Rietveld and Kiverstein, 2014). According to Bruineberg and Rietveld (2014), the concept of a “*landscape* of affordances” aims to capture the interrelatedness of the available affordances. “*Affordances are not encountered as a set of separate possibilities for action, but rather as a nested structure of interrelated affordances*” (p. 3) (Bruineberg and Rietveld, 2014). That is, as suggested by Seifert et al. (2017), skilled climbers appear to perceive a cluster of holds rather than multiple separate holds, suggesting that they perceive one continuous (prospective) opportunity for action.

Further to the longer relative durations of stationary ascent (that we hypothesized to be dedicated to finding the route path), the D route also resulted in a higher number of performatory movements and a shorter relative duration in the performatory state in comparison with the H route. These findings might be due to the fact that there were twice as many possible actions (i.e., dual grasping) offered by the D route holds. Indeed, knowing that each respective route was composed of 20 holds, one might expect that at least 20 performatory movements would occur for each route. However, < 20 performatory movements occurred in the H route, which is partly explained by some falls that occurred early in practice, but also, more importantly, because some climbers skipped some of the holds later in practice. Conversely, even when falls are included, the climbers exhibited more than 20 performatory movements on the V and D routes, suggesting that they used dual-hand grasping on one hold. Dual-hand grasping is likely to be commensurate with stationary states and could lead to less fluid actions (i.e., increased jerk) and indicate a lack of *use-ability* (for instance in comparison to a crossed arm movement; Boschker and Bakker, 2002). This point of interpretation reinforces the interpretation that the learners climbed hold-by-hold in more complex routes rather than fluent ascending actions.

### Effect of practice and transfer of learning

The results showed a positive effect of practice, as the learners significantly improved the performance outcome (i.e., less falls and shorter ascent duration) and climbing fluency (i.e., lower jerk coefficient) through the four climbs across all routes designs (see Cordier et al., 1994; Seifert et al., 2014a; Orth et al., 2017b). Performance outcomes and climbing fluency were also significantly worse on the transfer test in comparison to the H route. However, there was no difference in total climb duration and climbing fluency between the transfer test and routes V and D. This confirms that climbing a route that invites easier and more conventional action modes (i.e., using horizontal hold grasping pattern) leads to enhanced performance outcomes and climbing fluency. Furthermore, our findings suggest that more practice than four sessions appears to be necessary to develop more complex action modes (i.e., using vertical hold grasping pattern) needed to climb routes that consisted of V and D holds. However, this hypothesis needs to be tested in a future study because the amount of practice was not manipulated in the current study.

To explain these changes of the performance outcome with practice, our main hypothesis was that exploratory, stationary, and performatory behaviors would decrease as learning occurs. With practice, the learners decreased the relative duration of hold exploration, suggesting that they improved their *perception of hold grasp-ability*; that is, they learnt which and how to grasp holds (as also reported by the PAE questionnaire), confirming previous findings (Seifert et al., 2015; Orth et al., 2017a). The absence of a significant difference in relative duration of exploration between the transfer test and the fourth session for the three other routes confirmed this trend.

The climbers also decreased the number of stationary states across learning sessions, suggesting that they became attuned to affordances that supported more fluent climbing (also described previously as improvement of “route finding” skill; see Cordier et al., 1993, 1994 for more details). However, performance on the transfer test led to higher number of stationary states and longer relative duration of stationary state than the fourth sessions of the three routes performed during practice. Taken together, the fact that the improvement of route finding skill and hold grasp-ability perception did not fully enhance performance on the transfer test suggests that the intermediate climbers in the current study might need more sessions of practice and/or sessions involving more variation (e.g., including routes mixing hold types) for a complete positive transfer to occur (for similar hypothesis, see Huet et al., 2011). Future research with higher amount of practice or manipulation of the amount of practice would be necessary to confirm this suggestion.

The number of performatory movements decreased as performance increased during learning sessions, confirming that participants' climbing efficacy improved as a function of practice. In addition, the perception of the route (from PAE questionnaire) also improved with practice suggesting that changes in the performatory movements might reflect *higher hold-use-ability*. The transfer test produced shorter relative durations dedicated to performatory movements than the fourth session of the three routes performed during practice. We interpreted this shorter relative duration dedicated to performatory movements as *lower hold use-ability* because it's associated with a higher number of performatory movements on the transfer test, V and D routes in comparison to H route.

In conclusion, our study emphasized that with practice, climbers learnt to explore as they improved their attunement to affordances and they enlarged their landscape of affordances; in particular, the climbers improved their route-finding skill, hold grasp-ability and use-ability. However, these improvements were dependent on the complexity of the route design as they persisted only on the H route and not the V route, the D route, and the transfer test, suggesting that the learners did not transfer skills to more complex settings. Thus, specific to considerations for the design of climbing practice conditions, these findings indicate that it is crucial to design holds and routes that mirror the complexity of outdoor climbing, but in a manner that invites effective and safe exploration. This is particularly the case in extreme sports such as climbing, whereby learners continuously perform in new, more challenging contexts.

## Author contributions

LS, DO, and BM contributed to the experimental design. LS and DO contributed to the data collection. LS, JB, and RH contributed to the data analysis. LS, DO, BM, RH, JB, and MD contributed to the interpretation of the results and the writing.

## Conflict of interest statement

The authors declare that the research was conducted in the absence of any commercial or financial relationships that could be construed as a potential conflict of interest.

## Footnotes

^1^Invariants are subsets of the spatio-temporal structure of light converging at the eyes, of sound at the ears, etc. which specify properties of the individual-environment system such as affordances.
